# Possibility for Visualizing the Muscle Microstructure by q-Space Imaging Technique

**DOI:** 10.1155/2022/7929589

**Published:** 2022-08-08

**Authors:** Yasushi Sera, Daisuke Nakashima, Junichi Hata, Hirotaka James Okano, Kazuki Sato, Masaya Nakamura, Takeo Nagura

**Affiliations:** ^1^Institute for Integrated Sports Medicine, Keio University School of Medicine, Shinjuku-ku, Tokyo, Japan; ^2^Department of Orthopaedic Surgery, Keio University School of Medicine, Shinjuku-ku, Tokyo, Japan; ^3^Division of Regenerative Medicine, The Jikei University School of Medicine, Minato-ku, Tokyo, Japan; ^4^Department of Clinical Biomechanics, Keio University School of Medicine, Shinjuku-ku, Tokyo, Japan

## Abstract

In the human body, skeletal muscle microstructures have been evaluated only by biopsy. Noninvasive examination of the microstructure of muscles would be useful for research and clinical practice in sports and musculoskeletal areas. The study is aimed at determining if q-space imaging (QSI) can reveal the microstructure of muscles in humans. Forty-three Japanese subjects (controls, distance runners, powerlifting athletes, and teenage runners) were included in this cross-sectional study. Magnetic resonance imaging of the lower leg was performed. On each leg muscle, full width at half maximum (FWHM) which indicated the muscle cell diameters and pennation angle (PA) were measured and compared. FWHM showed significant positive correlations with PA, which is related to muscle strength. In addition, FWHM was higher for powerlifting, control, distance running, and teenager, in that order, suggesting that it may be directing the diameter of each muscle cell. Type 1 and type 2 fibers are enlarged by growth, so the fact that the FWHM of the control group was larger than that of the teenagers in this study may indicate that the muscle fibers were enlarged by growth. Also, FWHM has the possibility to increase with increased muscle fibers caused by training. We showed that QSI had the possibility to depict noninvasively the microstructure like muscle fiber type and subtle changes caused by growth and sports characteristics, which previously could only be assessed by biopsy.

## 1. Introduction

The human muscles are divided into two types of skeletal muscle fibers: fast and slow [1]. Generally, fast fibers are considered to provide instantaneous power, whereas slow fibers provide high endurance. In humans, muscle fiber types are classified as types I, IIa, and IIx, also called type IIb fiber; these types vary in muscle cell diameter size, amount of mitochondria, etc., in addition to the myosin heavy chain (MyHC) [2]. Muscle activity is influenced by muscle cell diameter, which correlates with muscle strength and decreases with age [3]. Therefore, visualization of the muscle cell diameter is important for analyzing the actual muscle activity and histological muscle character.

Previous studies have shown that muscle cell diameter and muscle fiber type in each muscle differ based on the type of sport [4, 5]. According to Fry's review, weightlifting and powerlifting athletes had enlarged type II fibers, and bodybuilders had enlarged type I fibers [4]. The study that examined the difference in muscle fiber type between the vastus lateralis and deltoid muscle by biopsy revealed less slow fibers in powerlifting athletes in both muscles [6]. Additionally, long-distance runners had a higher percentage of slow fibers in the vastus lateralis than in the deltoid muscle [6]. In a study from a non-sport-specific perspective, it has been reported that muscle cell size generally increases after birth and decreases with age, and particularly type II muscle cell size increases with resistance exercises [7]. As mentioned above, many studies have reported the change in muscle cell diameter, but no conclusions have been reached with regard to the types of changes in the muscle fiber. Komi and Vitasalod reported that the proportion of muscle fiber types is genetically determined [8]. Metaxas et al. published that muscle fiber types could change with training [9]. Also, regardless of muscle fiber types, muscle architecture is important, and muscle cross-sectional area (CSA) and pennation angle (PA) are known to be increased by resistance training [10].

Additionally, the disease concept of “sarcopenia,” which is defined as aging-related loss of muscle mass [11], has attracted attention recently. Muscular weakness with aging is mainly caused by decrease in fast muscle (type II fiber) [12], and slow muscle (type I fiber) decreases due to muscle atrophy associated with paralysis and disuse syndrome, such as in patients with congestive heart failure [13]. By clarifying the fine structure of muscles, such as muscle cell diameter and fiber type, research that investigates the differences in muscle properties due to characteristics of sport and aging is expected to develop, resulting in the development of more specific training methods. Clinical evaluation of muscle microstructure has commonly been performed by biopsy. Consequently, in recent years, muscle studies have often excluded children, and studies on adults are increasing because of the invasiveness of biopsy. As an alternative method of biopsy, muscle fiber type identification methods using electromyography, bioelectrical impedance analysis, and magnetic resonance imaging (MRI) have been developed [14–17]. However, no method other than biopsy has been established yet. Recent studies have used MRI to try to analyze the muscle structure. Diffusion tensor imaging (DTI) has also been suggested to be related to muscle strength [18]. T2 mapping is typically used for cartilage quality evaluation, which reflects only water content [19].

Peng et al. reported an evaluation of muscle fiber type by spin-lattice relaxation time in the rotating frame (T1*ρ*) mapping [15]. T1*ρ* reflects the water content like T2 mapping and the apparent diffusion coefficient (ADC) and is used for cartilage and tumor evaluation [20]. When performing T1*ρ* imaging and biopsy in rats and humans, the T1*ρ* parameters and muscle fiber type changes are similar, and differences have been found between the T1*ρ* values in the tibialis anterior muscle (TA) and soleus muscle (SOL) [15]. Although this method may lead to the prediction of muscle fiber type by the volume of collagen, it becomes invalid when the collagen density changes because of dehydration. Alternatively, q-space imaging (QSI) is a quantitative diffusion-weighted imaging (DWI) procedure that enables the detection of delicate changes in the microstructure of environments in which free water movement is restricted [21]. The movement of water molecules in tissues with limited diffusion can be evaluated by QSI [22], and it has been reported that QSI has a high microstructure resolution [23–25]. Additionally, QSI can provide additional diffusion values, including the full width at half maximum (FWHM) (*μ*m) [26, 27]; this is obtained from the shapes of the probability density function (PDF). Previously, the usefulness of QSI for the diagnosis of multiple sclerosis and some kinds of cancer has been reported [28, 29]. In the field of muscle research, the possibility of using QSI to clarify the muscle fiber direction has been reported [30]. We have previously reported that QSI can visualize the distribution of fast and slow muscle fibers similar to immunohistological staining of muscle by distinguishing the difference in cell diameters among muscle fiber types in an animal study [31]. It also shows that QSI can assess minor muscle edema due to muscle fatigue [32].

In the future, an invasive procedure for diagnosing muscle quality, such as a biopsy, would be difficult on ethical grounds. Hence, the development of a noninvasive method for assessing muscle quality is awaited. The study is aimed at determining if QSI could reflect muscle microstructure such as cell diameters and, consequently, reflect the quality and structure of muscle characteristics resulting from sports characteristics and growth.

## 2. Methods

### 2.1. Study Population

This study included a total of 43 Japanese volunteers: 12 healthy adults without healthy exercise habits who have confirmed by self-report at this time that they have no lifestyle-related diseases or history of serious trauma (control group), 10 elite distance runners, 11 elite powerlifting athletes, and 10 teenage track and field athletes. Cases were sampled by self-selection. The control, distance runners, and powerlifting groups were all males, and the teenage group included 3 boys and 7 girls. The control and distance running groups were recruited from Japanese university students. These two groups were selected without differences between them, except for their sports characteristics. They had the healthy lifestyle and eating habits of typical college students. The powerlifting group consisted of high-level adult males, and their eating habits consisted mainly of a high-protein diet. The teenagers were selected from the general Japanese teenage population and had general healthy lifestyles and eating habits. The powerlifting group had older competitors, which was thought to be due to competition characteristics. The sample size calculation was estimated to be 9–11 people per group (36–46 total), which would provide 80% power with alpha equal to 0.05. This study was approved by the Keio University's Ethics Committee (No. 20170024) and conducted in accordance with the Declaration of Helsinki and subsequent amendments and equivalent ethical guidelines. The contents of the study were explained to all participants, and informed consent was obtained.

### 2.2. Physical Measurement

**Figure 1:**
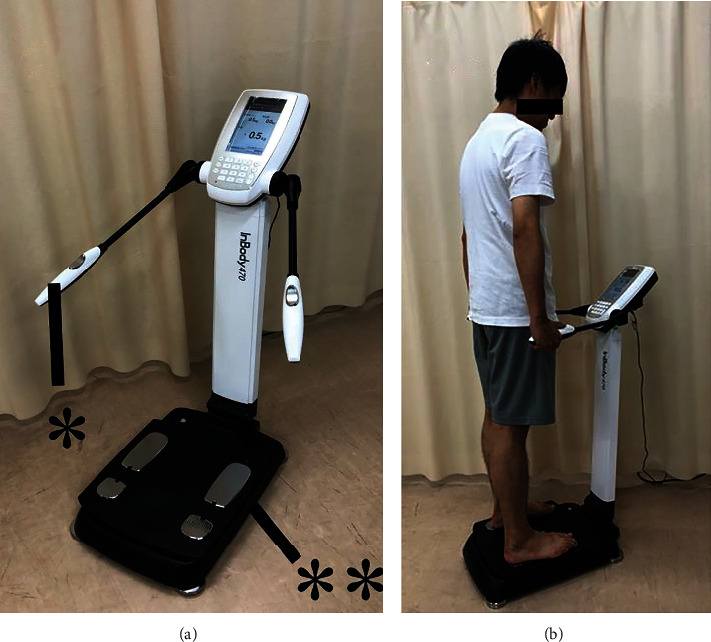
Method for measuring body composition. (a) Image of the InBody 470®; ∗: hand electrodes; ∗∗: foot electrodes. (b) Measurement of body composition.

The participants' height and weight were measured. The body fat percentage and skeletal muscle mass index (SMI) were measured using a body composition analyzer that used a direct segmental multifrequency bioelectrical impedance analysis method (InBody 470®; InBody Japan Inc., Tokyo, Japan) (Figures 1(a) and 1(b)). These raw data are summarized in the Supplemental Table.

### 2.3. MRI Protocol

All participants underwent imaging of both lower legs via a 3.0 T MRI scanner (Magnetom Skyra fit 3T; Siemens Healthineers, Erlangen, Germany) with a 30-channel Body Coil (Siemens Healthineers). Axial T2-weighted images (T2WI) were acquired for reference images of the muscles. DWI including DTI and QSI (using pulsed gradient spin echo) were performed. Details of the MRI protocol are shown in Table 1.

### 2.4. Imaging Calculation

DTI and QSI were analyzed by using an in-house program (developed in C++; Embarcadero Technologies, Inc., Austin, TX, USA).

The detailed diffusion values and their calculation procedures were as previously described [26, 33, 34].

The radial diffusivity (RD), one of the parameters of DTI, based on the conventional monoexponential model was calculated from a part of the QSI data (*b* value, 0, and 800 s/mm^2^). The RD was calculated according to the following formula:

(1) $$\begin{array}{c} \text{RD}=\frac{\lambda 2+\lambda 3}{2}. \end{array}$$

Based on the hypothesis that QSI can more precisely aid the investigation of the muscle structure than DTI, we calculated the following RD for QSI in addition to the above RD for DTI.

The non-Gaussian probability density function (PDF) of water diffusion for QSI was obtained by a Fourier transformation of the data based on the Stejskal–Tanner diffusion preparation [35]. The full width at half maximum (FWHM (*μ*m)) of the PDF was calculated [36]. We performed the tensor calculation by acquiring the six-axis data of QSI (Table 1). In this study, we defined a unique radial FWHM based on the concept of radial anisotropy by the Gaussian diffusion model to evaluate anisotropy. Radial FWHM was used to indicate the cross section of muscle cells exhibiting a spindle-shaped morphology, with regard to radial anisotropy according to the Gaussian diffusion model [36]. The three eigenvalues (*λ*1, *λ*2, *λ*3) and their corresponding eigenvectors were calculated. The radial FWHM was calculated using only two eigenvectors (*λ*2 and *λ*3) of the three eigenvalues (*λ*1, *λ*2, *λ*3), similar to the concept of RD in DTI.

The radial FWHM was calculated as follows:

(2) $$\begin{array}{c} \text{Radial FWHM}=\frac{\lambda 2+\lambda 3}{2}. \end{array}$$

The eigenvalues (*λ*2, *λ*3) in formula (2) are calculated using a different method than the eigenvalues (*λ*2, *λ*3) used in the DTI-RD formula (1), as explained above.

### 2.5. Imaging Analysis

**Figure 2:**
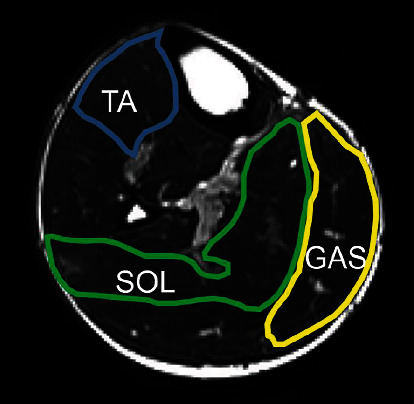
Methods for measuring the ROIs. The ROIs of the tibialis anterior muscle (blue square), soleus muscle (green square), and medial head of gastrocnemius muscle (yellow square) on a T2-weighted image. TA: tibialis anterior muscle; SOL: soleus muscle; GAS: gastrocnemius muscle.

TA and the medical head of gastrocnemius muscle (GAS) were identified by T2WI axial images at the maximum circumference of the lower legs. Major blood vessels were avoided, and a region of interest (ROI) was set entire circumference of each muscle by 2 researchers (Figure 2). Three measurements were taken to calculate the average value, and the left and right values were averaged. Each value was measured by using ImageJ ver. 1.53 software (available at http://rsbweb.nih.gov/ij/), and color mapping used Mango ver. 4.1 software (available at http://rii.uthscsa.edu/mango). Fiber reconstruction and tracking were performed using the Diffusion Toolkit version 0.6.4 software (available at http://trackvis.org/). DTI has been tracked with the following conditions: mask threshold between 0.02 and 1.23 and angle threshold is 30. The tracking data was used for TrackVis ver. 0.6.1 software (available at http://trackvis.org/). The muscle fascia identified in each muscle and fibers through the two planes were tracked. The images were measured five times using ImageJ for the pennation angle, and the average value was calculated.

### 2.6. Statistical Analysis

The data were summarized in Supplemental Table 1. Analysis was performed by using SPSS version 24® software (IBM Corp., Armonk, NY). For the measurement data, the mean and SD were calculated. Analysis of variance (ANOVA) and Dunnett's test were performed for comparisons of each imaging and physical strength parameter among the 4 groups. In addition to the above, we compared the correlation between the parameters of MRI and physical measurement using Spearman's rank correlation coefficient. Statistical significance was set at *P* = 0.05 for all tests.

## 3. Results

### 3.1. Results of Physical Measurement

First, we compared the body composition, and the results are presented in Table 2. Compared with the control group, the age, body weight, and SMI were significantly higher in the powerlifting group and significantly lower in the teenager group (*P* < 0.05). Body weight and SMI were in order of heaviness the powerlifting, control, distance running, and teenager groups.

### 3.2. Results of Magnetic Resonance Imaging Parameters among the Four Groups

**Figure 3:**
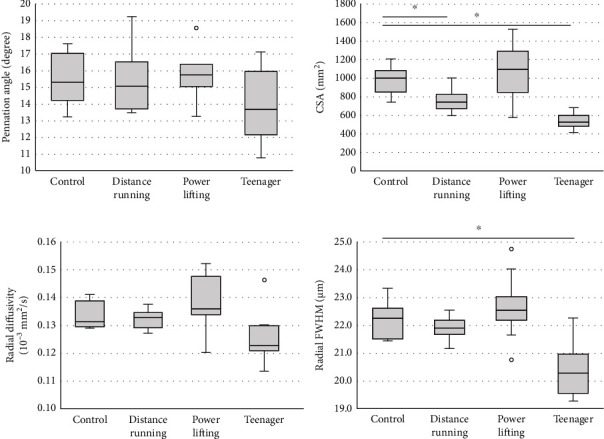
Quantification of magnetic resonance imaging parameters in the tibialis anterior muscle. Box and whisker plots: the bottom and top of the box are the first and third quartiles. The band inside the box is the second quartile (median). The ends of the whiskers represent the minimum and maximum of all of the data. White circles are outliers. CSA: cross-sectional area; FWHM: full width at half maximum. ^∗^*P* < 0.05 (Dunnett's test; reference = control).

**Figure 4:**
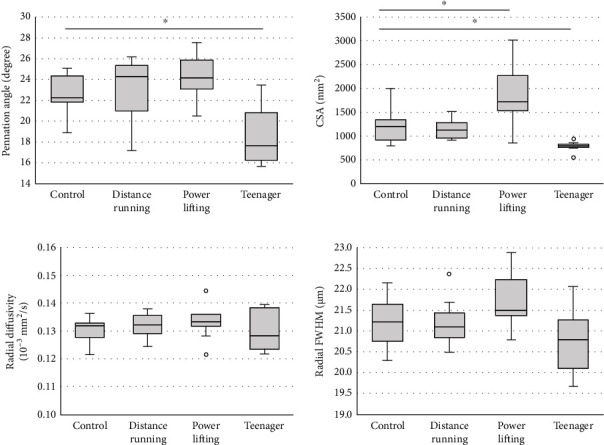
Quantification of magnetic resonance imaging parameters in the gastrocnemius muscle. Box and whisker plots: the bottom and top of the box are the first and third quartiles. The band inside the box is the second quartile (median). The ends of the whiskers represent the minimum and maximum of all of the data. White circles are outliers. CSA: cross-sectional area; FWHM: full width at half maximum. ^∗^*P* < 0.05 (Dunnett's test; reference = control).

**Figure 5:**
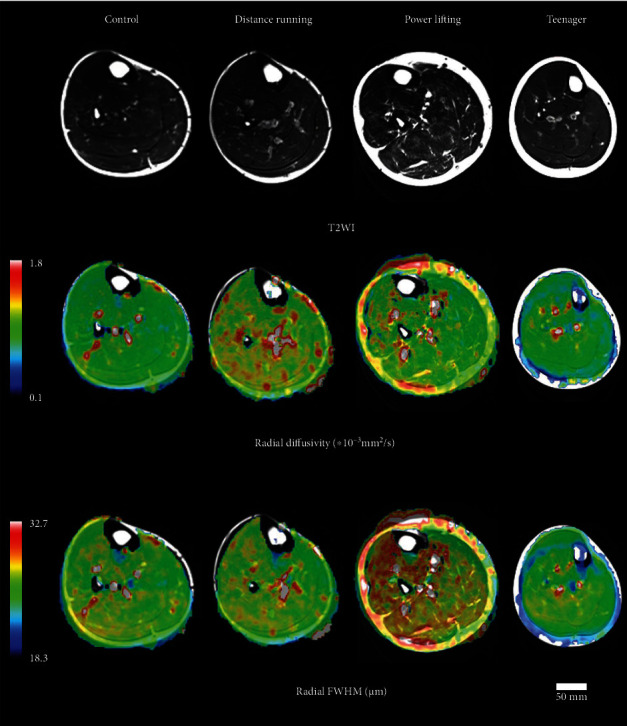
Color mapping axial sections of magnetic resonance imaging for the four groups. T2WI: T2-weighted imaging; FWHM: full width at half maximum.

Next, we compared the MRI results among the 4 groups. The MRI data are quantitatively presented in Figures 3 and 4. There were significant differences in the CSA, the RD, and the radial FWHM in the TA and GAS. Interestingly, the radial FWHM was significantly higher in the control group than in the teenager group. Representative T2WI images, RD on T2WI, and radial FWHM on T2WI are shown in Figure 5.

### 3.3. Correlations among the Parameters of Magnetic Resonance Imaging and Physical Measurement

Finally, we compared the correlation between the parameters of MRI and physical measurement. The radial FWHM of TA and GAS showed a moderate positive correlation with SMI, PA, and CSA. The RD of TA and GAS also showed a positive correlation with SMI and CSA (Tables 3 and 4). SMI, CSA, and radial FWHM were greater for the powerlifting, control, distance running, and teenager groups in that order.

## 4. Discussion

In this study, there were no significant differences in body weight and SMI between the control group and the distance runner group. On the other hand, the appearance of the powerlifting group was significantly higher in age, weight, PFB, and SMI than the control group. The teenager group was younger than the other groups and had lower height and SMI. The 4 groups in this study had different characteristics.

According to the theory of DTI, water molecules distribute by unrestricted diffusion. On the other hand, QSI is based on the theory that water molecules distribute according to restricted diffusion [19, 22]. In the case of muscle cells, water molecules are under restricted diffusion due to cell walls, collagen fiber, and mitochondria. The more detailed cell structures introduced by these factors can potentially be revealed by QSI [25]. In our previous study, we were able to distinguish between TA and SOL by using the FWHM in mice [31]. In this study, we used the radial FWHM, which is calculated from the short axis directions, *λ*2 and *λ*3, and thus represents the plane perpendicular to the muscle fiber direction, which is thought to contribute to the muscle cross-sectional area. We used RD calculated by DTI for the same reason. The radial FWHM in the teenager group was significantly lower than that in the control group in TA. Additionally, in TA and GAS, we observed a trend toward greater CSA and radial FWHM in the powerlifting, control, distance running, and teenager groups, in order. Previous studies have reported that muscle strength is positively correlated with muscle CSA, which is also consistent with the fact that SMI showed a similar trend [37]. In color mapping, the radial FWHM shows more differences from muscle to muscle than radial diffusivity, visualizing differences in muscle characteristics (Figure 5). In the present study, SOL was also analyzed, but no certain trend was observed in radial FWHM or other parameters since, unlike TA and GAS, SOL is a crossed fiber and was difficult to analyze. PA is mainly measured by ultrasound and MRI; however, measuring the same area is difficult in both methods. Additionally, differences were found between the control group and distance running groups, but both TA and GAS showed the highest PA in the powerlifting group and the lowest PA in the teenager group, which is consistent with previous reports that PA is related to muscle strength and CSA. Radial FWHM showed significant positive correlations with SMI, which indicates total body muscle mass, PA, which is related to muscle strength, and muscle CSA. Additionally, RD showed significant correlations with SMI and CSA, but the correlation coefficient was smaller than that of radial FWHM, suggesting that radial FWHM is more related to other parameters than RD. In previous reports, the number of muscle fibers does not change, the muscle mass is affected by the muscle cell diameters, and it was considered that the radial FWHM reflects the muscle cell diameter [7]. The larger radial FWHM also had a larger CSA, which may have correlated with SMI and PA. Type 1 and type 2 fibers are enlarged by growth, so the fact that the radial FWHM of the control group was larger than that of the teenagers in this study may indicate that the muscle fibers were enlarged [7]. It is predicted that the radial FWHM will increase with increased muscle fibers caused by training in the future.

In this study, we measured the powerlifting athletes to represent a group with many fast muscle fibers and distance runners to represent a group with many slow muscle fibers [4]. Reportedly, muscle fibers change in animals other than humans [38], but the number of muscle fibers remains unchanged from an early age. However, this is partly due to the low number of muscle fibers that can be obtained in a human needle biopsy, which is difficult to evaluate. Previous reports have noted that the type of muscle fiber changes in humans, but this topic has no consensus [39]. In a study of teenage youth soccer players, the percentage of muscle fiber types slightly varied with age, but all types of muscle fibers became larger and sprint performance improved [9, 40]. Whether this result is due to age or training remains unknown. However, muscle research from young and old, male and female, and athletes to the general public is expected to become even more important in the future. Previously, the comparison of muscle cell diameters has been performed by biopsy, but the application of this QSI is expected to noninvasively measure muscle cell diameters. Whether the observed differences in muscle fibers obtained in our groups were due to birth differences or training differences remains unclear. However, these children will train in the future, and how their muscle fiber changes can be measured will be assessed; thus, determining if the muscle cell diameter only increases or the muscle fiber type changes will be possible. This study showed that QSI could noninvasively reveal differences in muscle fiber types in humans and is expected to be used in many fields, such as training methods and sports injury and disability prevention.

This study has several limitations. First, we were unable to directly evaluate the muscle tissue, such as a muscle cell diameter and muscle fiber types, by biopsy. We have already reported a comparison of muscle tissue and QSI in mice, and we believe that direct comparison by muscle biopsy is required in the future in humans as well. Second, we used QSI to examine the average ROI values, so we evaluated fast and slow muscles together. Generally, fast muscles have a larger muscle cell diameter than slow muscles, but it is difficult to evaluate whether the muscle cell diameter is large or there are many fast muscles. We used QSI technique in this research, and validation of FWHM requires needle biopsy of the muscles; however, it is invasive to perform with healthy volunteers. The ratio of fast and slow muscles is used in assessments of training methods. Noninvasive determination of muscle fiber types and measuring muscle cell diameters are expected to be used not only in sports areas but also for the prevention of muscle atrophy and sarcopenia in the elderly. In conclusion, QSI could noninvasively depict the microstructure like muscle fiber type and subtle changes caused by growth and sports characteristics, which previously could only be assessed by biopsy.

## Figures and Tables

**Table 1:** Summary of the magnetic resonance imaging protocol.

Contrast	T2WI	DWI
Sequence	Rapid acquisition with relaxation enhancement	Pulsed gradient spin echo
Repetition time (ms)	6310	4000
Echo time (ms)	101	93
Average	2	1
Field of view (mm^2^)	400 × 275	400 × 275
Matrix size	512 × 352	128 × 88
Pixel resolution (mm^2^)	0.78	3.12
Slice thickness (mm)	4	8
Imaging time	1 min 47 sec	4 min 0 sec
Diffusion information		
Diffusion direction	6	
Δ/*δ* (ms)	45.6/27.9	
*b* value (s/mm^2^)	0, 50, 200, 450, 800, 1250, 1800, 2400, 3150, 4000	
*q* value (cm^−1^)	0, 59.1, 118.1, 177.2, 236.3, 295.3, 354.4, 409.2, 468.8, 528.3	

T2WI: T2-weighted imaging; DWI: diffusion-weighted imaging; Δ: diffusion gradient separation (time between the two leading edges of the diffusion gradients); *δ*: diffusion gradient duration.

**Table 2:** Summary of research participants.

	Control	Distance running	Powerlifting	Teenager
Number of subjects	12	10	11	10
Age^∗^	21.6 ± 1.1	21.4 ± 1.2	27.8 ± 10.3^∗∗^	13.0 ± 0.0^∗∗^
Height (cm)^∗^	171.3 ± 7.2	169.5 ± 4.1	166.5 ± 6.0	161.7 ± 5.6^∗∗^
Weight (kg)^∗^	64.1 ± 9.8	57.1 ± 4.3	86.5 ± 23.4^∗∗^	49.2 ± 6.1^∗∗^
Percent body fat (%)^∗^	17.0 ± 4.9	13.5 ± 3.4	24.6 ± 9.9^∗∗^	16.4 ± 6.1
Skeletal mass index (kg/m^2^)^∗^	7.80 ± 0.73	7.28 ± 0.28	9.64 ± 1.11^∗∗^	6.28 ± 0.63^∗∗^

^∗^ANOVA; ^∗∗^*P* < 0.05 (Dunnett's test; reference = control).

**Table 3:** Summary of the correlation between muscle parameters and magnetic resonance imaging parameters of the tibialis anterior muscle.

	SMI	Pennation angle	CSA	Radial diffusivity	Radial FWHM
SMI	1.00				
Pennation angle	0.38^∗^	1.00			
CSA	0.77^∗∗^	0.41^∗∗^	1.00		
Radial diffusivity	0.63^∗∗^	0.28	0.40^∗∗^	1.00	
Radial FWHM	0.78^∗∗^	0.40^∗∗^	0.71^∗∗^	0.75^∗∗^	1.00

SMI: skeletal muscle mass index; CSA: cross-sectional area; FWHM: full width at half maximum. ^∗^*P* < 0.05 and ^∗∗^*P* < 0.01 (Spearman's rank correlation coefficient.).

**Table 4:** Summary of the correlation between muscle parameters and magnetic resonance imaging parameters of the gastrocnemius muscle.

	SMI	Pennation angle	CSA	Radial diffusivity	Radial FWHM
SMI	1.00				
Pennation angle	0.62^∗∗^	1.00			
CSA	0.89^∗∗^	0.53^∗∗^	1.00		
Radial diffusivity	0.36^∗^	0.28	0.29^∗∗^	1.00	
Radial FWHM	0.59^∗∗^	0.32^∗∗^	0.53^∗∗^	0.54^∗∗^	1.00

SMI: skeletal muscle mass index; CSA: cross-sectional area; FWHM: full width at half maximum. ^∗^*P* < 0.05 and ^∗∗^*P* < 0.01 (Spearman's rank correlation coefficient.).

## Data Availability

The data used to support the findings of this study are included within the supplementary information file.

## Supplementary Materials

Supplementary Materials Supplemental Table 1 is the raw result data. Results of physical measurements and magnetic resonance imaging parameters.

## References

[1] Pette D., Staron R. S. (2000). Myosin isoforms, muscle fiber types, and transitions. *Microscopy Research and Technique*.

[2] Sjogaard G. (1982). Capillary supply and cross-sectional area of slow and fast twitch muscle fibres in man. *Histochemistry*.

[3] Nilwik R., Snijders T., Leenders M. (2013). The decline in skeletal muscle mass with aging is mainly attributed to a reduction in type II muscle fiber size. *Experimental Gerontology*.

[4] Fry A. C. (2004). The role of resistance exercise intensity on muscle fibre adaptations. *Sports Medicine*.

[5] Serrano N., Colenso-Semple L. M., Lazauskus K. K. (2019). Extraordinary fast-twitch fiber abundance in elite weightlifters. *PLoS One*.

[6] Tesch P. A., Karlsson J. (1985). Muscle fiber types and size in trained and untrained muscles of elite athletes. *Journal of Applied Physiology*.

[7] Verdijk L. B., Snijders T., Drost M., Delhaas T., Kadi F., van Loon L. J. C. (2014). Satellite cells in human skeletal muscle; from birth to old age. *Age*.

[8] Komi P. V., Vitasalo J. H. (1976). Signal characteristics of EMG at different levels of muscle tension. *Acta Physiologica Scandinavica*.

[9] Metaxas T. I., Mandroukas A., Vamvakoudis E., Kotoglou K., Ekblom B., Mandroukas K. (2014). Muscle fiber characteristics, satellite cells and soccer performance in young athletes. *Journal of Sports Science and Medicine*.

[10] Franchi M. V., Atherton P. J., Reeves N. D. (2014). Architectural, functional and molecular responses to concentric and eccentric loading in human skeletal muscle. *Acta Physiologica*.

[11] Cruz-Jentoft A. J., Baeyens J. P., Bauer J. M. (2010). Sarcopenia: European consensus on definition and diagnosis: report of the European working group on sarcopenia in older people. *Age and Ageing*.

[12] Lexell J., Sjostrom M., Nordlund A. S., Taylor C. C. (1992). Growth and development of human muscle: a quantitative morphological study of whole vastus lateralis from childhood to adult age. *Muscle & Nerve*.

[13] Vescovo G., Serafini F., Facchin L. (1996). Specific changes in skeletal muscle myosin heavy chain composition in cardiac failure: differences compared with disuse atrophy as assessed on microbiopsies by high resolution electrophoresis. *Heart*.

[14] Kramer I. F., Snijders T., Smeets J. S. J. (2017). Extensive type II muscle fiber atrophy in elderly female hip fracture patients. *The Journals of Gerontology. Series A, Biological Sciences and Medical Sciences*.

[15] Peng X. G., Wang Y., Zhang S. (2017). Noninvasive assessment of age, gender, and exercise effects on skeletal muscle: initial experience with T1*ρ* MRI of calf muscle. *Journal of Magnetic Resonance Imaging*.

[16] Uchiyama T., Nakayama T., Kuru S. (2017). Muscle development in healthy children evaluated by bioelectrical impedance analysis. *Brain & Development*.

[17] Naddaf E., Milone M., Mauermann M. L., Mandrekar J., Litchy W. J. (2018). Muscle biopsy and electromyography correlation. *Frontiers in Neurology*.

[18] Hata J., Nagata H., Endo K. (2015). Assessment of human skeletal muscle contraction and force by diffusion tensor imaging. *Open Journal of Radiology*.

[19] Juras V., Bohndorf K., Heule R. (2016). A comparison of multi-echo spin-echo and triple-echo steady-state T2 mapping for in vivo evaluation of articular cartilage. *European Radiology*.

[20] Wheaton A. J., Dodge G. R., Elliott D. M., Nicoll S. B., Reddy R. (2005). Quantification of cartilage biomechanical and biochemical properties via T1*ρ* magnetic resonance imaging. *Magnetic Resonance in Medicine*.

[21] Callaghan P. T., Coy A., MacGowan D., Packer K. J., Zelaya F. O. (1991). Diffraction-like effects in NMR diffusion studies of fluids in porous solids. *Nature*.

[22] Budzik J. F., Balbi V., Verclytte S., Pansini V., Thuc V. L., Cotten A. (2014). Diffusion tensor imaging in musculoskeletal disorders. *Radiographics*.

[23] Nakashima D., Fujita N., Hata J. (2020). Quantitative analysis of intervertebral disc degeneration using Q-space imaging in a rat model. *Journal of Orthopaedic Research*.

[24] Callaghan P. T. (1996). NMR imaging, NMR diffraction and applications of pulsed gradient spin echoes in porous media. *Magnetic Resonance Imaging*.

[25] Katsura M., Suzuki Y., Hata J. (2014). Non-Gaussian diffusion-weighted imaging for assessing diurnal changes in intervertebral disc microstructure. *Journal of Magnetic Resonance Imaging*.

[26] Jensen J. H., Helpern J. A., Ramani A., Lu H., Kaczynski K. (2005). Diffusional kurtosis imaging: the quantification of non-gaussian water diffusion by means of magnetic resonance imaging. *Magnetic Resonance in Medicine*.

[27] Fujiyoshi K., Hikishima K., Nakahara J. (2016). Application of q-space diffusion MRI for the visualization of white matter. *The Journal of Neuroscience*.

[28] Yamada I., Hikishima K., Miyasaka N. (2015). Esophageal carcinoma: evaluation with q-space diffusion-weighted MR imaging ex vivo. *Magnetic Resonance in Medicine*.

[29] Tanikawa M., Nakahara J., Hata J. (2017). *q* -space myelin map imaging for longitudinal analysis of demyelination and remyelination in multiple sclerosis patients treated with fingolimod: a preliminary study. *Journal of the Neurological Sciences*.

[30] Taylor E. N., Hoffman M. P., Aninwene G. E., Gilbert R. J. (2015). Patterns of intersecting fiber arrays revealed in whole muscle with generalized Q-space imaging. *Biophysical Journal*.

[31] Hata J., Nakashima D., Tsuji O. (2019). Noninvasive technique to evaluate the muscle fiber characteristics using q-space imaging. *PLoS One*.

[32] Nakashima D., Hata J., Sone Y. (2021). Detecting mild lower-limb skeletal muscle fatigue with stimulated-echo q-space imaging. *Magnetic Resonance in Medical Sciences*.

[33] Assaf Y., Ben-Bashat D., Chapman J. (2002). High b-value q-space analyzed diffusion-weighted MRI: application to multiple sclerosis. *Magnetic Resonance in Medicine*.

[34] Farrell J. A., Smith S. A., Gordon-Lipkin E. M., Reich D. S., Calabresi P. A., van Zijl P. C. M. (2008). High b-value q-space diffusion-weighted MRI of the human cervical spinal cord in vivo: feasibility and application to multiple sclerosis. *Magnetic Resonance in Medicine*.

[35] Stejskal E. O., Tanner J. E. (1965). Spin diffusion measurements: spin echoes in the presence of a time-dependent field gradient. *The Journal of Chemical Physics*.

[36] Basser P. J. (1995). Inferring microstructural features and the physiological state of tissues from diffusion-weighted images. *NMR in Biomedicine*.

[37] Lexell J., Taylor C. C., Sjostrom M. (1988). What is the cause of the ageing atrophy?: total number, size and proportion of different fiber types studied in whole vastus lateralis muscle from 15- to 83-year-old men. *Journal of the Neurological Sciences*.

[38] Loughna P. T., Izumo S., Goldspink G., Nadal-Ginard B. (1990). Disuse and passive stretch cause rapid alterations in expression of developmental and adult contractile protein genes in skeletal muscle. *Development*.

[39] Schiaffino S. (2010). Fibre types in skeletal muscle: a personal account. *Acta Physiologica*.

[40] Metaxas T., Mandroukas A., Michailidis Y., Koutlianos N., Christoulas K., Ekblom B. (2019). Correlation of fiber-type composition and sprint performance in youth soccer players. *Journal of Strength and Conditioning Research*.

