# Supplementary material for: Possibility for Visualizing the Muscle Microstructure by q-Space Imaging Technique
Source: Appl Bionics Biomech. 2022 Aug 8;2022:7929589. doi: 10.1155/2022/7929589 (PMC9377983; doi:10.1155/2022/7929589)
Supplement: Supplementary Materials — Supplemental Table 1 is the raw result data. Results of physical measurements and magnetic resonance imaging parameters. [file 7929589.f1.pdf]

type	Age	PBF(%)	SMI	Height	Weight	PA_TA	PA_GAS	PA_SOL	Area_TA	Area_GAS	Area_SOL	mDTI_RD_TA	mDTI_RD_GAS	mDTI_RD_SOL	mHW_RD_TA	mHW_RD_GAS	mHW_RD_SOL	mDTI_RD_TA	mDTI_RD_GAS	mDTI_RD_SOL
Control	21	13	9	165	67.3	15.6507	24.6875	26.8312	1086.9141	1988.0371	2194.0919	0.001398041	0.001337856	0.001412141	23.19821418	21.46302668	22.78159829	0.1398041	0.1337856	0.1412141
Control	21	15.9	7.8	180	67.1	14.1657	24.5133	25.6552	741.9434	798.3398	1229.9805	0.001388154	0.001280773	0.001349579	21.4515231	20.7748559	21.46713652	0.1388154	0.1280773	0.1349579
Control	22	28.9	8.1	165	71.9	17.0431	21.7591	28.0218	859.1309	1230.957	1791.0155	0.001319798	0.001330508	0.001421697	22.61393174	22.15368544	22.77889767	0.1319798	0.1330508	0.1421697
Control	24	19.6	8	171	68	16.9722	25.0965	23.4461	1077.6367	1339.5996	1883.0567	0.001385357	0.001325129	0.001434295	23.34874406	22.00210748	23.03122056	0.1385357	0.1325129	0.1434295
Control	21	11.8	7.4	179.8	58.2	13.2491	22.3043	27.1259	830.078	1172.1192	1631.5918	0.001296274	0.001274226	0.001350525	21.48141675	21.09370786	22.59717846	0.1296274	0.1274226	0.1350525
Control	23	18	8.4	181.5	75.9	14.9719	22.4597	21.6953	1003.174	1351.3183	1703.1249	0.001334561	0.001363082	0.001434139	22.63441588	21.70106907	23.28885876	0.1334561	0.1363082	0.1434139
Control	21	13.9	7.4	170	56.1	17.5202	22.0956	24.1472	1131.1034	1168.2129	1908.6914	0.001289796	0.001216542	0.001330076	22.42317325	20.74937864	22.51442016	0.1289796	0.1216542	0.1330076
Control	20	16.4	8.3	171.5	66.9	14.5183	23.9645	23.8633	1020.9962	1521.9725	2231.6893	0.001302895	0.001320859	0.001378815	21.90042027	21.32261283	22.56052742	0.1302895	0.1320859	0.1378815
Control	22	22	8.5	176	79.6	17.6196	22.1551	27.5549	1208.2519	1263.4278	1965.8203	0.001294117	0.001215625	0.001336681	22.40839023	20.65927868	22.54029045	0.1294117	0.1215625	0.1336681
Control	21	11.9	6.5	160	45.5	16.2929	22.2584	26.9704	851.8067	813.2325	1513.9161	0.00130612	0.001325883	0.001334845	21.52536872	21.33811756	21.26475514	0.130612	0.1325883	0.1334845
Control	21	18.6	6.8	162.5	54.1	13.9403	18.9116	25.7741	891.3574	920.8983	1285.6445	0.001291159	0.001308228	0.00142405	21.52753773	21.00056268	22.39950464	0.1291159	0.1308228	0.142405
Control	22	13.7	7.4	173	59	14.3427	21.7405	20.6425	1002.4414	909.912	1697.5098	0.001411343	0.001313425	0.001449185	22.08463871	20.29647353	22.92972074	0.1411343	0.1313425	0.1449185
Distance running	22	11.7	7.4	168	56.9	13.7467	24.7334	26.3229	599.1212	950.9278	1262.4512	0.001289744	0.001310032	0.001387854	21.83407979	21.1001626	22.49169986	0.1289744	0.1310032	0.1387854
Distance running	21	9.4	7.4	171	54.1	15.3172	25.375	25.9961	729.4921	1520.2637	1538.3299	0.001325794	0.001342204	0.001443005	22.15187064	20.85794922	22.33718744	0.1325794	0.1342204	0.1443005
Distance running	19	12.6	7.1	164	52.9	16.1132	23.82	22.2083	680.9084	915.5273	1169.9219	0.001361538	0.001373662	0.001454054	22.54402043	22.37004353	23.23089345	0.1361538	0.1373662	0.1454054
Distance running	21	17.3	7.4	178	66.3	14.257	21.78	22.8069	999.7559	1042.2364	1567.3826	0.001375329	0.001379684	0.001431005	22.0519334	20.79524503	22.59339876	0.1375329	0.1379684	0.1431005
Distance running	22	18.3	6.7	171	55.7	13.4727	18.5742	28.7385	810.3029	1119.8731	1219.9707	0.001322264	0.001350553	0.001386764	21.16706064	21.03886469	21.44677199	0.1322264	0.1350553	0.1386764
Distance running	20	11.3	7.3	165	54.9	19.2305	23.9664	26.3497	715.8203	1131.1034	1521.2403	0.001332068	0.001332711	0.00145959	21.56525047	21.0916545	22.82463237	0.1332068	0.1332711	0.145959
Distance running	23	11.1	7.2	166	53.1	15.0001	17.203	23.6986	833.0079	1249.2675	1549.0723	0.001335696	0.001243589	0.00130958	21.98296274	21.68472793	22.18426818	0.1335696	0.1243589	0.130958
Distance running	22	15.4	7.2	169	57.1	17.779	26.1498	24.4274	654.7852	956.7872	1391.8456	0.00134094	0.001276638	0.001373113	21.82157265	20.47490099	22.04734699	0.134094	0.1276638	0.1373113
Distance running	22	17.9	7.3	173	62.4	13.583	25.3522	30.8452	824.4629	1161.377	1408.4472	0.001290992	0.001295022	0.001426614	22.24100133	21.15895221	22.78635995	0.1290992	0.1295022	0.1426614
Distance running	22	9.8	7.8	170	58	15.1294	24.5955	26.7042	750.0001	1385.9863	1790.283	0.001271936	0.001310967	0.001357133	21.72229674	21.34883496	21.72742437	0.1271936	0.1310967	0.1357133
Power lifting	32	35.6	10.5	163	103.7	16.1193	23.8541	25.9261	1067.871	1730.4687	2467.7736	0.001522575	0.001317713	0.001356068	24.74258574	21.36852764	22.03843412	0.1522575	0.1317713	0.1356068
Power lifting	21	11.8	9.1	160	63.5	13.2579	24.8969	26.4561	842.7733	1529.0528	1987.5489	0.001360233	0.001326425	0.001414133	22.37765634	21.47108272	22.53970451	0.1360233	0.1326425	0.1414133
Power lifting	21	26.9	9.1	171	86.9	15.7356	27.5398	27.5883	1095.459	1713.3789	2259.2773	0.001414029	0.001326429	0.001418427	22.99369365	20.81995478	22.17365361	0.1414029	0.1326429	0.1418427
Power lifting	26	12.3	8.7	170	69.1	13.4257	25.0788	26.9212	702.8808	1125.9765	1241.9434	0.00137584	0.001334343	0.001490808	22.37313687	21.41881461	22.94026162	0.137584	0.1334343	0.1490808
Power lifting	47	38.2	11.2	169	114	18.5826	25.9664	29.3636	1528.8088	3016.1133	2973.877	0.001481584	0.001444736	0.001400291	24.02337403	22.24199714	21.66592811	0.1481584	0.1444736	0.1400291
Power lifting	38	37.8	11.5	178	136.7	15.4213	22.0633	25.7293	1213.379	2273.9257	3160.1563	0.001476889	0.001443731	0.001393995	22.54701965	22.45846303	21.38171731	0.1476889	0.1443731	0.1393995
Power lifting	20	22.6	8.9	168	77.6	15.7544	23.094	25.7629	1285.8886	1705.8105	2317.3827	0.001344924	0.001338422	0.00141846	22.17904958	21.48570372	22.22268234	0.1344924	0.1338422	0.141846
Power lifting	42	23	10.5	161	85.9	15.0721	25.8731	25.8223	1444.8241	2524.414	2215.8204	0.001202409	0.001215447	0.001189182	23.04108922	22.87764108	22.27597129	0.1202409	0.1215447	0.1189182
Power lifting	22	14.4	9	169.5	69.7	18.6451	24.1762	24.4939	1294.9219	1797.3633	1958.2518	0.0013378	0.00133332	0.001405002	22.73606175	21.51385024	22.49491612	0.13378	0.133332	0.1405002
Power lifting	19	29.9	9.4	165	84.6	15.5284	24.0081	24.9125	929.4434	1812.9883	2015.6249	0.0012871	0.001281572	0.001323921	21.65586969	20.78662721	21.34981625	0.12871	0.1281572	0.1323921
Power lifting	18	18.4	8.1	157	59.7	16.3743	20.5445	23.6123	577.8808	856.6896	1119.6289	0.001356001	0.001359658	0.001478389	20.76915162	22.00623697	23.14926852	0.1356001	0.1359658	0.1478389
Teenager	13	12	6.8	161	48.3	15.6065	16.4202	21.4689	498.7793	820.8009	1212.6464	0.001300651	0.001238197	0.001321774	21.59315248	20.7178792	22.11797454	0.1300651	0.1238197	0.1321774
Teenager	13	13.4	7.3	157	51.5	12.4501	22.1733	27.8939	497.8027	798.0955	1571.289	0.001464359	0.00139096	0.001400178	22.25632481	22.07050301	22.51479049	0.1464359	0.139096	0.1400178
Teenager	13	12.7	5.7	163	42.7	10.7879	17.7404	17.4425	590.0879	546.1425	875.4883	0.001135136	0.001219485	0.001320782	19.28367223	19.81658279	21.16039643	0.1135136	0.1219485	0.1320782
Teenager	13	25.7	6.2	163	56.3	17.132	15.7707	20.6361	416.7479	940.6738	1226.0741	0.001228062	0.001253084	0.001348211	20.16984848	20.18343024	21.91441938	0.1228062	0.1253084	0.1348211
Teenager	13	16.9	5.4	160	42.9	13.6222	20.3302	18.5535	593.0176	753.1738	1217.5293	0.001228789	0.001215777	0.001315155	19.53509377	19.66904029	21.88300536	0.1228789	0.1215777	0.1315155
Teenager	13	6.8	6.9	173	51.1	14.8734	23.4978	17.3226	440.6738	767.0899	1074.2188	0.001297772	0.001394873	0.001483608	20.74165923	21.1912397	22.19727233	0.1297772	0.1394873	0.1483608
Teenager	13	21.3	6	159	50.7	13.7672	15.6823	21.454	566.1622	854.0039	1188.9648	0.001235886	0.001293485	0.001376905	20.1606065	21.08310941	22.41368567	0.1235886	0.1293485	0.1376905
Teenager	13	20	5.8	162	46.9	12.2046	16.7778	19.0614	600.83	770.7522	1105.4688	0.001209977	0.001270622	0.001385405	20.38135199	20.71990189	22.41277349	0.1209977	0.1270622	0.1385405
Teenager	13	23.5	6.8	167	60.1	17.0529	19.3568	23.7775	678.7108	811.5234	1233.1543	0.001206577	0.001297233	0.001333793	19.55502129	20.855767	21.84459347	0.1206577	0.1297233	0.1333793
Teenager	13	11.5	5.9	152	41.3	12.0812	17.5512	18.4863	494.1405	790.2833	1132.5683	0.001229021	0.001382518	0.0014378	20.46159578	21.51823867	22.53926726	0.1229021	0.1382518	0.14378
